# Exclusionary states in older age and their temporary effects on cognitive decline

**DOI:** 10.1186/s40359-025-02574-7

**Published:** 2025-03-18

**Authors:** Georgios Pavlidis

**Affiliations:** 1 Department of Social and Psychological Studies, Karlstad University, Universitetsgatan 2, Karlstad, 65188 Sweden; 2 Institution of Culture and Society, Linkoping University, Bredgatan 33, Norrköping, 60221 Sweden

**Keywords:** Episodic memory, Social relations, Exclusion, Loneliness

## Abstract

**Background:**

Exclusion from social relations (ESR) describes severe states of social isolation in older age that may be associated with poorer cognitive outcomes. Previous studies on cognitive aging provide mixed evidence for the effects of social isolation and loneliness in shaping cognitive outcomes among older adults. In addition, the joint consideration of social isolation and loneliness remains rarely used in the empirical examination of cognitive aging, whereas an exclusionary perspective is missing.

**Methods:**

Using a sample (*N* = 7,830) from the Survey of Health, Ageing, and Retirement in Europe (SHARE), this study examined the cross-sectional and longitudinal effects of three ESR states in older age (*ESR and lonely*, *ESR but not lonely*, *not ESR but lonely*) on episodic memory. Living alone or without a partner, being active in the labor market, and social participation were also included as exclusionary states in linear mixed models with health, demographics, and socioeconomic factors as covariates.

**Results:**

Cross-sectionally, ESR states in older age are associated with worse episodic memory independent of loneliness. There was no evidence for longitudinal effects between ESR states at baseline and episodic memory slopes over time.

**Conclusions:**

It was concluded that the negative effects of loneliness-typified ESR states on cognitive aging may be temporary and reversible, as a function of older adults’ transition in-and-out of these exclusionary states.

**Supplementary Information:**

The online version contains supplementary material available at 10.1186/s40359-025-02574-7.

## Background

The demographic change into an aging population has brought global attention to older adults’ welfare and social integration. In this context, social isolation and loneliness are considered public health concerns that are associated with worse health outcomes [1–3] and mortality [4]. In addition, loneliness and social isolation have been associated with adverse cognitive decline trajectories [5], with significant implications for theory, policy, and clinical practice. Two well cited theories that have been used to explain the links between social isolation, loneliness, and cognitive decline are the neuro-endocrinological reaction to loneliness theory [6, 7] and the cognitive enrichment hypothesis [8].

According to the *neuro-endocrinological reaction theory*, loneliness is a negative feeling that stems from social isolation and leads to increases in the activation of the hypothalamic pituitary adrenocortical axis affecting the hippocampus and other brain areas known to be related to memory [6, 7]. The *cognitive enrichment hypothesis* is an alternative theory that emphasizes the instrumental properties of social isolation and the lack of cognitive stimulation among socially isolated individuals. This theory postulates that an enriched social engagement in older age constitutes an in-vivo cognitive stimulation that buffers against the expected cognitive deterioration with aging [8]. In contrast, socially isolated older persons may live in a cognitively impoverished milieu that further precipitates cognitive aging, which may increase their risk for dementia onset [9].

Social isolation and loneliness are often conflated both in the public discourse [10] and in clinical practice [11], as they describe related, yet distinct aspects of relational deficits in older age. In social sciences [12], medicine [1], and social epidemiology [13], social isolation refers to objective shortages in functional (e.g., receiving support) and structural aspects (e.g., network size) that typify older adults’ social networks. Unlike the objective attributes of social isolation, loneliness in these fields refers to the subjective perception of relational deficits, defined as a negative feeling stemming from either social isolation, or one’s unfulfilled intimacy within existing social networks [1, 11–13]. Social isolation and loneliness are weakly to moderately correlated in quantitative studies [14], because some socially isolated older adults may not feel lonely, while others may feel lonely “in the crowd” [12]. However, both social isolation and loneliness have their own clinical significance in assessing cognitive decline with aging [15].

In the present study, the cross-sectional and longitudinal associations of social isolation, loneliness and cognitive decline with aging were examined from an exclusionary perspective, i.e., by examining severe states of social isolation in older age. Exclusion from social relations (ESR) is a kindred concept to social isolation that has been recently used to make important distinctions in the objective assessment of relational deficits in older age [16]. The main thesis of this concept is that scoring zero in some objective measure of social network size is an exclusionary state that is qualitatively distinct from that of having a small social network (see also Umberson & Karas Montez [3]).

In that sense, older “network-less” adults may be more disadvantaged than those with a small social network at an older age (see also Litwin & Levinsky [17]), and these disadvantages may be disproportionally greater than those observed between older adults with small and large social networks. Foster et al. [4] used a similar exclusionary perspective and concluded that a threshold exists when linking deficits in social relations with mortality, with those living alone and rarely visited by friends and family having a 77% increased mortality risk. In the cognitive aging literature, similar distinctions have been applied for exclusionary states such as living alone [18, 19], widowhood or divorce [20], and retirement [21].

**Fig. 1:**
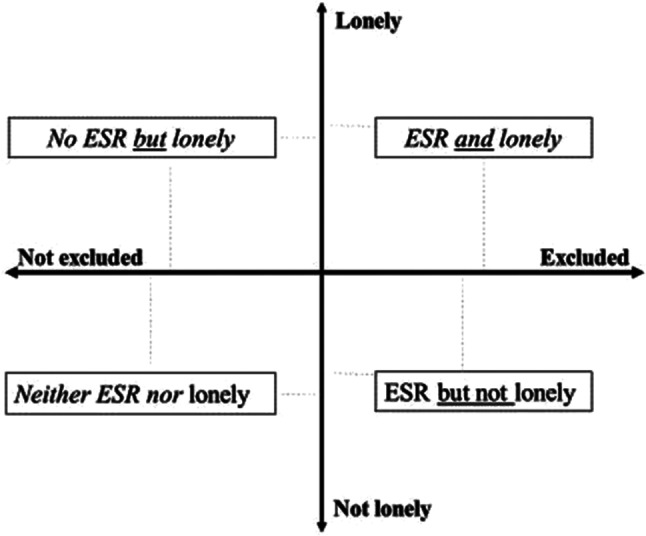
States of exclusion from social relations (ESR) in older age based on measures of social isolation and loneliness. Note ESR = states of exclusion from social relations, namely scoring zero in network size; lonely are those who feel lonely sometimes or all the time, not lonely those who never feel lonely. This graph has been also published in Pavlidis [25]

Accordingly, ESR in older age is used in this study to describe severe relational deficits, whereas loneliness is used to distinguish three different expressions of exclusionary states (i.e., ESR and lonely, ESR not lonely, not ESR but lonely; see Fig. 1). Newall and Menec [22] argued that these three exclusionary states represent older adults who face different challenges. Relevant research indicates increased levels of psychological distress among older adults that are in ESR states *but do not feel* lonely, when compared with those who are *neither* in ESR states *nor* lonely [23]. However, these differences were disproportionately smaller than those observed between the *neither ESR nor lonely* and the *ESR and lonely* groups, as well as between the *neither ESR nor lonely* and the *not ESR but lonely* groups of older adults. Similar findings have been reported for the associations of ESR with older adults’ quality of life [24] and depressive symptomatology [16].

Normal cognitive aging (i.e., typical cognitive decline trajectories) refers to age-related declines in cognitive performance that are not typified by cognitive impairments (e.g., dementia). Among the cognitive domains that are sensitive to typical age-related cognitive declines with aging, episodic memory is of particular interest due to its moderate correlation with functional ability [26–28]. Studies on social isolation in older age have indicated its robust longitudinal associations with typical memory decline [29, 30], while most studies on loneliness and typical cognitive decline highlight a negative association between the two constructs [31]. However, Boss and colleagues [31] noted in their systematic review of the relevant literature that the available evidence is mixed; and the correlations of loneliness and typical cognitive performance often turn to non-significant when other demographic and psychosocial risk factors are considered in tandem. In two meta-analyses, Lara and colleagues [5] and Harrington and colleagues [32] concluded that loneliness is associated with an increased risk of dementia and with a decreased global cognitive function. In both meta-analyses, however, the lack of sufficient data and the heterogeneity of the methodology used in the reviewed studies did not make it feasible to explore (in aggregation) the longitudinal associations of loneliness with specific cognitive domains.

To date, only a limited number of studies on cognitive aging considered ESR and loneliness in tandem [15, 33]. In a relevant study, McHugh and colleagues [15] reported that older adults who (given their social isolation status) were less lonely than expected had better memory performance than those who (given their social isolations status) were lonelier than expected. Similarly, Lara and colleagues [33] reported that loneliness in older age, but not social isolation, is associated with poorer episodic memory performance. However, neither of these studies [15, 33] identified statistically significant associations of loneliness and social isolation with the rates of cognitive decline with aging. In contrast, Yu et al. [34] argued that social isolation, but not loneliness, is associated with steeper cognitive decline rates in older age.

As per previous studies on cognitive decline with aging [15, 33], education, depression, financial distress, and overall health status were included as significant covariates with considerable effects on older adults’ cognitive vitality. Living alone, living without a partner, retirement, and social participation were also included under the scope of examination for the effects of ESR states on cognitive decline, as all have been associated with worse cognitive outcomes [18–21, 35]. It was hypothesized that:

- H1. Living alone or without a partner, not being active in the labor market, less social participation, and ESR states that are typified by loneliness, are independently associated with worse episodic memory in older age.
- H2. Living alone or without a partner, not being active in the labor market, less social participation, and ESR states that are typified by loneliness, are independently associated with increased rates of episodic memory decline.

## Method and material

### Study population and design

This study is based on longitudinal data from the fourth, sixth, and eighth waves of the Survey of Health, Ageing and Retirement in Europe (SHARE), which were collected in 2011, 2015, and 2019/20 respectively [36]. As the largest social science panel study in Europe, SHARE administered a detailed social network module in the fourth, sixth, and eighth waves [37]. Twelve of the participating countries (Austria, Germany, Sweden, Spain, Italy, France, Denmark, Switzerland, Belgium, the Czech Republic, Poland, Slovenia) administered the cognitive function module in addition to the social network module in these waves. Information about the procedures of the SHARE survey (e.g., sampling method, data collection method, and response rates), including ethical approval in all countries, can be found in Bergmann et al. [37] and the official website of the survey.

### Participants

The sample consisted of non-institutionalized adults older than 50 years and younger than 81 years (the average life expectancy at birth in the European population [38]). In the fourth wave of the SHARE, 33,694 non-institutionalized participants in this age group were administered the social network and the cognitive function modules, as well as a questionnaire about loneliness. The sample used in this study consisted of 7.830 older adults who provided complete information (complete data) on all the variables examined in this analysis in all three waves under examination (i.e., waves four, six, and eight). Among them, 40.4% were male and 59.6% female, with the mean age in the fourth wave being *M* = 60.96, *SD* = 5.87. The characteristics of the sample across the three time-points of measurement can be found in Table 1.

### Measures

#### Episodic memory

Episodic memory was assessed in SHARE using a ten common nouns list for the immediate and a delayed recall task [36]. The respondents heard the list once and were asked to recall the items in any order immediately (immediate recall), as well as at the end of the cognitive function module (delayed recall). As per previous research [39] and given the strong correlations between the immediate and delayed recall scores in the SHARE data (in this study and across waves *r* =.661 −.738, *p* <.001), the raw number of correctly recalled words in both tasks were summed, with the aggregated score ranging from zero to 20.

#### Loneliness

The short version of the Revised University of California at Los Angeles Loneliness scale (R-UCLA [40]) was used in SHARE to measure loneliness. The scale consists of three items asking about loneliness indirectly, namely the frequency of feeling a lack of companionship, being left out, and isolation from others, with three available responses: hardly ever or never, some of the time, and often. The final score is a summation of the three items and the aggregated score ranges between 3 and 9. Cronbach’s α for the loneliness scale across the three waves was *a* = 0.752 − 0.922, with higher scores indicating higher levels of loneliness. As per previous research [41], loneliness was dichotomized into those who had *not felt lonely* at all (score = 3) and those who had *felt lonely* sometimes or almost always (scores = 4–9).

#### Living alone and living with a partner

The participants in the SHARE study were asked whether any other person was living in their household and the number of household members [36]. Participants reporting zero cohabitants were coded as *living alone*. The participants were also asked to state whether they were married and cohabiting with their partner; were in a registered partnership; were married but not cohabiting with their partner; had never been married; were divorced; or were widowed. The participants were coded as living *with a partner* if they stated that they were married and cohabiting with their partner or were in a registered partnership.

#### Depression

Depression was a dichotomized variable stemming from the EURO-D 12-item scale used in SHARE to assess depressive symptomatology [36]. The scale uses 12 binary yes/no response items to yield a total score between 0 and 12, with the items covering depression, pessimism, suicidality, guilt, sleep, interest, irritability, appetite, fatigue, concentration, enjoyment, and tearfulness during the last month. Scores of four or higher are consistent with the clinical manifestation of depression [42]. Because the EURO-D used in SHARE reflects a breadth of depressive symptoms that may not co-exist, no Cronbach alpha is calculated. However, the EURO-D used in SHARE has been validated in a previous study [43] as being internally consistent, capturing the essence of its parent instrument.

**Table 1:** Descriptive analysis of demographics, EURO-D, R-UCLA scores, financial distress, as well as health and social network variables in the three waves of the study

	Wave 4		Wave 6		Wave 8	
	Mean	SD	Mean	SD	Mean	SD
Age	60.96	5.87	64.77	5.86	69.46	5.86
Years of education	11.45	4.45				
SoPa	2.06	2.45	2.112	2.53	2.08	2.48
Episodic memory _(aggregated)_	10.51	3.24	10.70	3.35	10.19	3.43
	%	%	%	%	%	%
Gender _(male/female)_	40.4	59.6				
Living with partner _(yes/no)_	71.4	28.6	68.5	31.5	65.2	34.8
Living alone _(yes/no)_	20.1	79.9	23.1	76.9	27.5	72.5
Depression _(yes/no)_	21.6	78.1	20.3	79.7	22.2	77.8
Gali _(yes/no)_	37.3	62.7	38.7	61.3	43.9	56.1
Active in labor _(yes/no)_	39.2	60.8	26.6	73.4	18.2	81.8
*Perceived health*	%		%		%	
Excellent	10.4		9.1		7.2	
Very good	25.6		23.3		21.5	
Good	39.8		42.0		42.4	
Fair	19.3		20.7		23.5	
Poor	4.9		4.7		5.3	
*Financial distress*						
Easily	35.4		45.6		42.5	
Fairly easily	32.6		28.1		34.2	
With some difficulty	24.3		19.6		18.4	
With great difficulty	7.7		6.7		4.9	
*ESR states*						
ESR not lonely	1.7		0.9		0.6	
ESR and lonely	1.0		1.2		0.7	
Neither ESR nor lonely	65.1		62.6		62.5	
Not ESR but lonely	32.2		35.3		36.2	

Note. M = mean, SD = standard deviation, R-UCLA = loneliness scale, SoPA = social participation, Gali = limitations due to health, ESR = states of exclusion from social relations, namely coring zero in network size; lonely are those with R-UCLA scores > 3, not lonely those with R-UCLA scores = 3

#### Social participation

Participants in SHARE were asked whether they have done voluntary or charity work; attended an educational or training course; gone to a sport, social, or other kind of club; and whether they took part in a political or community-related organization. They were also asked about their participation frequency in these activities on a Likert scale, with the possible responses of almost every day, almost every week, almost every month, and less often [36]. For this study, participants’ responses were reverse-scored and summed, so that higher frequency scores represented more social participation (range = 0–16). Since the social participation index reflected a breadth of activities that may not co-exist (i.e., one may participate in volunteering but not in political or community organizations), no Cronbach alpha was calculated for this measure.

#### Social network size and States of exclusion from social relations (ESR States)

The participants in SHARE were asked: “Over the last 12 months, who are the people with whom you most often discussed important things? These people may include your family members, friends, neighbors, or other acquaintances”; with the instruction to name up to six adults. In addition, respondents were given the opportunity to list an additional adult that was important for them for any other reason [36]. As per previous research [24, 25], the network size variable was dichotomized, with participants scoring zero in this inventory coded as being in an objective ESR state. Those who reported one person or more in their network were coded as being embedded in a social network (hence, as no ESR). In extension, dummy coding variables representing different expressions of ESR states across loneliness levels (i.e., *not felt lonely* or *felt lonely*) were created (i.e., ESR and lonely, ESR but not lonely, not ESR but lonely; reference category neither ESR nor lonely).

#### Other covariates

Education was an imputed variable representing the number of years attending full-time education (range = 0–25), with higher values indicating more years of education. Financial distress was assessed by asking participants about the degree of difficulty the household has to make ends meet, with the available responses being easily, fairly easily, with some difficulty, with great difficulty. These responses were coded (range = 1–4) with higher values indicating greater difficulty. Perceived health was measured by asking participants: “Would you say your health is.,” with the available responses given in a 5-point Likert scale ranging from “poor” to “excellent”. These responses were reverse coded (range = 1–5) with higher values indicating worse health. Limitations in activities people usually do because of a health problem were measured with a single item using the probe: “For the past six months or more, have you been limited in activities people usually do because of a health problem?”, with the potential answers being *limited / not limited*. The participants were also asked about their employment status, with the possible responses being retired; employed or self-employed; unemployed; permanently sick; a homemaker. Those reporting being employed or self-employed were coded as *active* in the labor market. More information about these variables (before coding) can be found in Bergmann et al. [37].

### Analysis plan

#### Attrition analysis

The data were analyzed using SPSS v.29. Independent sample t-test was used to compare the mean age, years of education, social participation, and the aggregated episodic memory scores (hereafter memory) between the participants present only in the fourth wave (initial sample) and those present in all three waves. Pearson’s chi-square test (*χ*^2^) was conducted to examine statistically significant differences in the distribution of the study’s categorical variables (gender, living alone, depression, living with partner, being limited by health issues, health, being active in the labor market, financial distress, and ESR states) between the participants present only in the fourth wave and those present in all three waves.

#### Main analysis

The cross-sectional and longitudinal associations of ESR states with memory were assessed using linear mixed models with maximum likelihood estimation (MLE) in five steps, with an unstructured covariance type for the estimation of random slopes, according to the instructions of Hox and colleagues [44]. The equation of this model can be expressed as:

undefined

$$\eqalign{{Y_{ij}} & = {\beta _0} + {\beta _1}{W_{ij}} + \sum \> _{k = 1}^K{\beta _{2k}}{X_{kij}} \cr & + \sum \> _{l = 1}^L{\beta _{3{\rm{ }}L}}{Z_{li}} + \sum \> _{k = 1}^K{\beta _{4k}}\left( {{W_{ij}} \times \>{X_{kij}}} \right) \cr & + \sum \> _{l = 1}^L{\beta _{5{\rm{ }}L}}\left( {{W_{ij}} \times {Z_{li}}} \right) + {b_{0i}} + {b_{1i}}{W_{ij}} + {\varepsilon _{ij}} \cr} $$

where *Y*_*i*j_ is the episodic memory for the participant *i* in the observation *j*, *β*_0_ the fixed intercept, *β*_1_ is the fixed effect of the different waves of measurement *W* for the participant *i* in the observation *j*, *β*_2*k*​_ are the fixed effects of the *X*_*k*_ time-varying predictors for the participant *i* at observation *j*, *β*_3*l*​_ are the fixed effects of the *Z*_*1*_ time-invariant predictors for the participant *i*, *β*_4*k*​_ are the fixed effects of the interactions between *W* and the time-varying predictors, *β*_5*l*​_ are the fixed effects of the interactions between *W* and the time-invariant predictors, *b*_0*i*_ is the random intercept for the participant *i*,* b*_1*i*​_ is the random slope for the participant *i* at wave *W* at the observation *j*, and ε_*i*w_ the residual error term. The three time-points (waves) of measurement were preferred over age as the time variable in the models because they offered better data visualization options. Instead, age at the fourth wave was used as the operationalization of the participants’ age. Since the dataset comprised of complete data, no imputation techniques were used for missing data.

In the first step, a null model (${Y_{iw}} = {\beta _0} + {b_{0i}} + {\varepsilon _{iw}}$) was constructed with memory as the outcome variable and scaled identity as the covariance type of fixed effects with an ordinal time-variable representing the three waves of measurement. This served as the baseline model to compare the Akaike’s Information Criterion (AIC) and the Schwarz’s Bayesian Criterion (BIC) fit indices with the subsequent models. In the second model, time (*β*_*1*_*W*) was included as a fixed effect covariate to examine whether memory declined across the three time-points of measurement (${Y_{iw}} = {\beta _0} + {W_{iw}} + {b_{0i}} + {\varepsilon _{iw}}$).

In the third model, the time-invariant predictors (i.e., age at the fourth wave, gender, years of education) and the time-variant predictors (living alone, financial distress, living with a partner, being active in the labor market, being limited by health issues, perceived health, depression, social participation) were included in the model. The dummy coding variables representing different expressions of ESR states across loneliness levels (i.e., ESR and lonely, ESR but not lonely, not ESR but lonely; reference category neither ESR nor lonely) were also included in the model as time-variant predictors.

$$\eqalign{{Y_{ij}} & = \beta {\>_0} + \beta {\>_1}{W_{ij}} + \sum \> _{k = 1}^K\beta {\>_{2k}}{X_{kij}} \cr & + \sum \> _{l = 1}^L\beta {\>_{3{\rm{ }}L}}{Z_{li}} + {b_{0i}} + {\varepsilon _{ij}} \cr} $$

undefined_0} + \beta {\>_1}{W_{ij}} + \sum \> _{k = 1}^K\beta {\>_{2k}}{X_{kij}} \cr & + \sum \> _{l = 1}^L\beta {\>_{3{\rm{ }}L}}{Z_{li}} + {b_{0i}} + {\varepsilon _{ij}} \cr} ]]>

In the fourth model, the time-variable was added as a random effect covariate to examine the variance of intercepts in memory scores across the three time-points of measurement (random intercept), the variance of memory scores between participants across the three time-points of measurement (random slope), and the covariance between the random intercept and random slope.

$$\eqalign{{Y_{ij}} & = \beta {\>_0} + \beta {\>_1}{W_{ij}} + \sum \> _{k = 1}^K\beta {\>_{2k}}{X_{kij}} \cr & + \sum \> _{l = 1}^L\beta {\>_{3{\rm{ }}L}}{Z_{li}} + {b_{0i}} + {b_{1i}}{W_{ij}} + {\varepsilon _{ij}} \cr} $$

undefined_0} + \beta {\>_1}{W_{ij}} + \sum \> _{k = 1}^K\beta {\>_{2k}}{X_{kij}} \cr & + \sum \> _{l = 1}^L\beta {\>_{3{\rm{ }}L}}{Z_{li}} + {b_{0i}} + {b_{1i}}{W_{ij}} + {\varepsilon _{ij}} \cr} ]]>

In the fifth step, the interaction terms for both the time-variant and the time-invariant predictor of the model with the time-variable *W* were included as fixed effects predictors, to examine whether these interaction terms can explain the variance in the memory slopes between the participants in the three time-points of measurement, over and above their cross-sectional effects.

$$\eqalign{{Y_{ij}} & = \beta {\>_0} + \beta {\>_1}{W_{ij}} + \sum\limits_{k = 1}^K {} \beta {\>_{2k}}{X_{kij}} \cr & + \sum\limits_{l = 1}^L {} \beta {\>_{3{\rm{ }}L}}{Z_{li}} + \sum\limits_{k = 1}^K {} \beta {\>_{4k}}\left( {{W_{ij}} \times \>{X_{kij}}} \right) \cr & + \sum\limits_{l = 1}^L {} \beta {\>_{5{\rm{ }}L}}\left( {{W_{ij}} \times \>{Z_{li}}} \right) + {b_{0i}} + {b_{1i}}{W_{ij}} + {\varepsilon _{ij}} \cr} $$

undefined_0} + \beta {\>_1}{W_{ij}} + \sum\limits_{k = 1}^K {} \beta {\>_{2k}}{X_{kij}} \cr & + \sum\limits_{l = 1}^L {} \beta {\>_{3{\rm{ }}L}}{Z_{li}} + \sum\limits_{k = 1}^K {} \beta {\>_{4k}}\left( {{W_{ij}} \times \>{X_{kij}}} \right) \cr & + \sum\limits_{l = 1}^L {} \beta {\>_{5{\rm{ }}L}}\left( {{W_{ij}} \times \>{Z_{li}}} \right) + {b_{0i}} + {b_{1i}}{W_{ij}} + {\varepsilon _{ij}} \cr} ]]>

## Results

### Attrition analysis

The results indicate that attrition (dropping out from the fourth to the eighth wave) was associated with older age (*t*(33692) = 31.977, *p* <.001), fewer years of education (*t*(33692) = -17.681, *p* <.001), less participation in social activities (*t*(33692) = 18.760, *p* <.001), and worse memory (*t*(33692) = -28.119, *p* <.001). Pearson’s chi-square tests (*χ*^2^) of independence indicated that being male (*χ*^*2*^(1) = 47.275, *p* <.001), being depressed (*χ*^*2*^(1) = 86.228, *p* <.001), being limited by health issues (*χ*^*2*^(1) = 161.051, *p* <.001), not being active in the labor market (*χ*^*2*^(1) = 455.336, *p* <.001), being in worse health (*χ*^*2*^(4) = 604.183, *p* <.001), having more financial difficulties (*χ*^*2*^(3) = 274.603, *p* <.001), and being ESR and lonely (*χ*^*2*^(3) = 16.005, *p* <.001) were associated with an increased likelihood of attrition (see Table S1 in the supplementary file).

### Main analysis

The first (base) model provided the fit indices *AIC* = 117180.81, *BIC* = 117205.00, -2LL = 117174.81. The second model provided an improvement (decline) in the fit indices (*AIC* = 117105.67, *BIC* = 117137.93, -2LL = 117097.67), where the time-variable was statistically significant and negative (*estimate* = − 0.160, *SE* = 0.003, *p* <.001).

In the third model, the introduction of the time-variable, the time-invariant (i.e., gender, years of education), and the time-variant predictors (i.e., age, living alone, financial distress, living with a partner, being active in the labor market, being limited by health issues, perceived health, depression, social participation, ESR states) yielded further improvements in the fit indices (*AIC* = 113408.11, *BIC* = 113553.03, -2LL = 113372.11). A later time-point of measurement (*estimate* = − 0.167, *SE* = 0.005, *p* <.001) remained a statistically significant predictor of worse memory scores (a full account of Model 3 is provided in Table S2 in the supplementary file). The fourth model provided further improvement in the model fit indices (*AIC* = 113370.24, *BIC* = 113531.26, -2LL = 113330.24), whereas a statistically significant variance emerged in the slopes of memory scores between participants across the three time-points of measurement (*estimate* = 0.310, *SE* = 0.060, *Wald Z* = 5.168, *p* <.001).

In the fifth model, the interaction terms of the predictors with the time-variable were introduced and further improved the fit indices of the model (*AIC* = 113254.90, *BIC* = 113528.63, -2LL = 113186.90). In this model, older age (*estimate* = − 0.027, *SE* = 0.010, *p* =.006), being male (*estimate* = 0.886, *SE* = 0.095, *p* <.001), fewer years of education (*estimate* = 0.125, *SE* = 0.011, *p* <.001), more financial distress (*estimate* = − 0.278, *SE* = 0.052, *p* <.001), not being active in the labor market (estimate = − 0.457, SE = 0.122, *p* <.001), worse perceived health (*estimate* = − 0.139, *SE* = 0.055, *p* =.011), being depressed (*estimate* = 0.420, *SE* = 0.118, *p* <.001), and less social participation (*estimate* = 0.105, *SE* = 0.019, *p* <.001) emerged as statistically significant predictors of worse memory scores. In addition, older adults who were *ESR and lonely* (estimate = -1.906, SE = 0.183, *p* <.001) or *ESR but not lonely* (estimate = − 0.914, SE = 0.391, *p* =.019) had statistically significant lower memory scores than those who were *neither ESR nor lonely*. Cross-sectionally, no other statistically significant associations emerged between the predictors and memory scores (see Table 2).

**Table 2:** Cross-sectional associations (Model 5) of memory scores with demographics, health, social participation, and States of exclusion from social relations

Parameter	Estimate	SE	df	t	Sig.	95% CI	
						Lower	Upper
Time	2.114	0.367	10281.119	5.757	< 0.001	1.394	2.833
Age ^(fourth wave)^	-0.027	0.010	9284.116	-2.760	0.006	− 0.046	− 0.008
Gender _(male/female)_	0.886	0.095	7863.142	9.309	< 0.001	0.700	1.073
Education	0.125	0.011	7870.861	11.778	< 0.001	0.104	0.146
Living alone _(yes/no)_	-0.045	0.161	10135.584	− 0.281	0.779	− 0.361	0.270
Financial distress	-0.278	0.052	11523.714	-5.375	< 0.001	− 0.380	− 0.177
Living with partner _(yes/no)_	0.124	0.144	9402.648	0.859	0.390	− 0.159	0.407
Active in labor _(yes/no)_	-0.457	0.122	13506.607	-3.745	< 0.001	− 0.696	− 0.218
Gali _(yes/no)_	-0.040	0.106	13167.268	− 0.381	0.703	− 0.249	0.168
Perceived health	-0.139	0.055	12283.352	-2.532	0.011	− 0.247	− 0.031
Depression _(yes/no)_	0.420	0.118	13005.818	3.550	< 0.001	0.188	0.652
Social participation	0.105	0.019	10892.869	5.471	< 0.001	0.067	0.142
ESR^1^							
ESR not lonely	-0.914	0.391	14244.309	-2.340	0.019	-1.680	− 0.149
ESR and lonely	-1.906	0.474	14219.573	-4.021	< 0.001	-2.835	− 0.977
Not ESR but lonely	-0.124	0.101	12975.292	-1.224	0.221	− 0.323	0.075

Note. SE = Standard error of means, df = degrees of freedom, CI = confidence intervals, Gali = limitations due to health, ESR = states of exclusion from social relations, namely coring zero in network size; lonely are those with R-UCLA scores > 3, not lonely those with R-UCLA scores = 3, R-UCLA = Revised UCLA loneliness scale

^1^ Reference group = neither ESR nor lonely

**Fig. 2:**
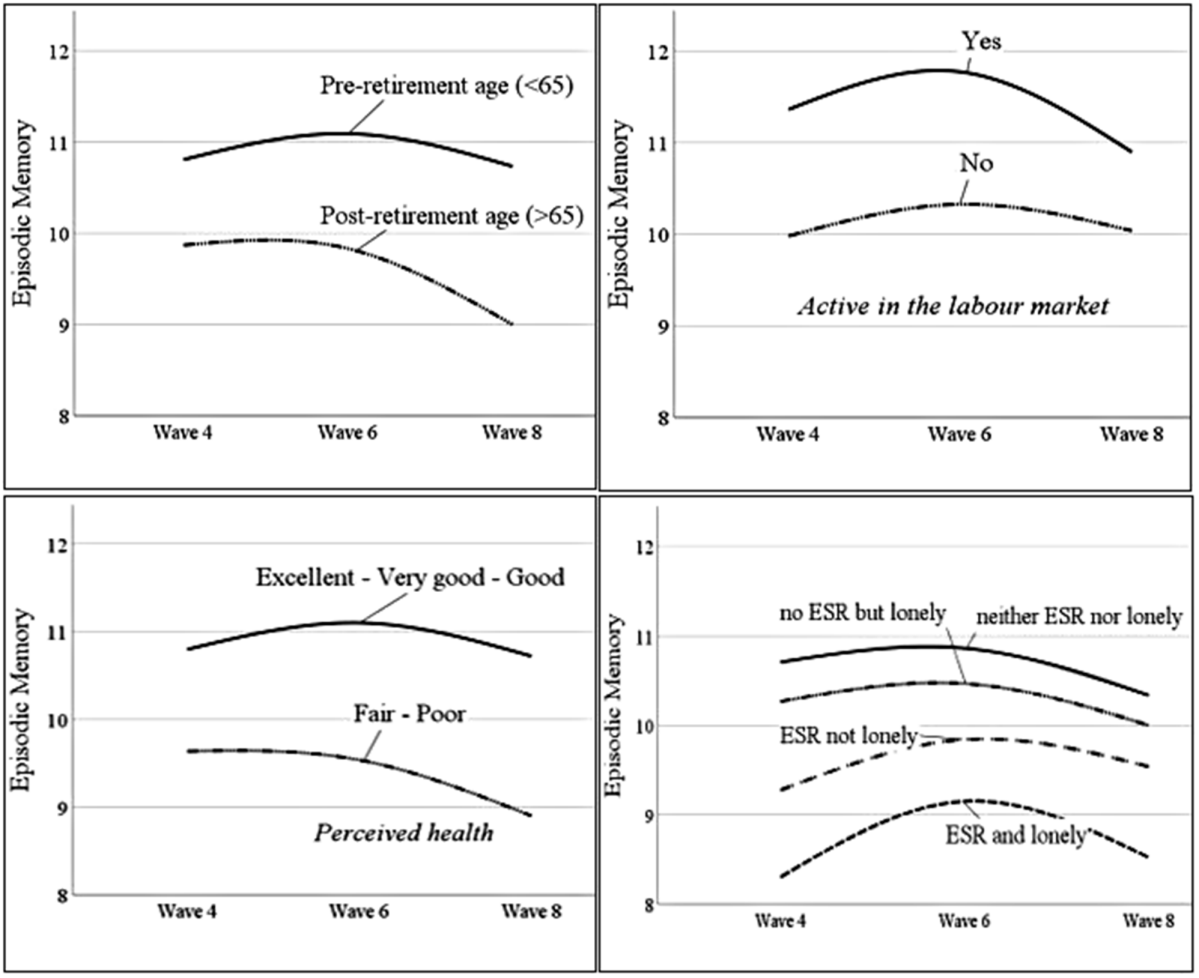
Trajectories of memory scores in older age across three waves of measurement, with age (top left), being active or not in the labor market (top right), perceived health status (bottom left), and ESR status (bottom right) as predictors. Note ESR = states of exclusion from social relations, namely scoring zero in network size; lonely are those with R-UCLA scores > 3, not lonely those with R-UCLA scores = 3

In the same model, older age (*estimate* = − 0.037, *SE* = 0.004, *p* <.001), being active in the labor market (*estimate* = 0.165, *SE* = 0.056, *p* =.003), and worse perceived health (*estimate* = − 0.085, *SE* = 0.025, *p* <.001) at the first time-point of measurement were statistically significant associated with higher rates of episodic memory decline rates (see Table 3). In addition, the participants who were in the *ESR and lonely* group in the fourth wave had lower rates of episodic memory decline compared to those who were *neither ESR nor lonely* in the same wave (*estimate* = 0.733, *SE* = 0.235, *p* =.002). A visual inspection of Fig. 2 indicates that the slower rates of episodic memory decline in the *ESR and lonely* group refers to an increase in memory scores between waves four and six. No other statistically significant longitudinal associations emerged between the predictors and the slopes of memory decline across the three waves of measurement.

**Table 3:** Longitudinal associations (Model 5) of memory slopes over three waves of measurement, with demographics, health, social participation, and States of exclusion from social relations as predictors

Parameter^1^	Estimate	SE	df	t	Sig.	95% CI	
						Lower	Upper
Age ^(fourth wave)^	-0.037	0.004	9828.136	-9.456	< 0.001	− 0.045	− 0.030
Gender _(male/female)_	0.038	0.040	7874.698	0.958	0.338	− 0.040	0.115
Education	-0.003	0.004	7859.796	− 0.695	0.487	− 0.012	0.006
Living alone _(yes/no)_	-0.034	0.071	10653.960	− 0.476	0.634	− 0.173	0.105
Financial distress	0.004	0.024	11843.304	0.162	0.871	− 0.043	0.051
Living with partner _(yes/no)_	-0.048	0.064	9754.672	− 0.754	0.451	− 0.173	0.077
Active in labor _(yes/no)_	0.165	0.056	16307.380	2.931	0.003	0.055	0.275
Gali _(yes/no)_	0.043	0.049	14085.696	0.891	0.373	− 0.052	0.139
Perceived health	-0.085	0.025	12972.536	-3.343	< 0.001	− 0.135	− 0.035
Depression _(yes/no)_	-0.018	0.055	13878.076	− 0.338	0.735	− 0.126	0.089
Social participation	0.010	0.009	11238.905	1.118	0.263	− 0.007	0.026
ESR^2^							
ESR not lonely	0.223	0.214	16115.303	1.043	0.297	− 0.197	0.643
ESR and lonely	0.733	0.234	15337.973	3.132	0.002	0.274	1.192
Not ESR but lonely	-0.020	0.047	13648.604	− 0.440	0.660	− 0.112	0.071

Note. SE = Standard error of means, df = degrees of freedom, CI = confidence intervals Gali = limitations due to health ESR = states of exclusion from social relations, namely scoring zero in network size; lonely are those with R-UCLA scores > 3, not lonely those with R-UCLA scores = 3, R-UCLA = Revised UCLA loneliness scale

^1^ Refers to the interaction term of the time-variable with the variables listed in the table (e.g., time x age).

^2^ Reference group = neither ESR nor lonely

Τhe Likelihood Ratio Test (LRT) was used to assess whether there was a statistical significant improvement in the nested models (Models 1–5). The results indicate that all subsequent models had a better model fit than their predecessors (*λ*^*1–2*^(1) = 77.140, *p <.001; λ*^*2–3*^ (14) = 3725.55, *p <.001; λ*^*3–4*^(2) = 41.866, *p <.001; λ*^*4–5*^(14) = 143.342, p <.001).

## Discussion

This study aimed to examine the cross-sectional and longitudinal associations of exclusionary states with cognitive decline in older age. It was hypothesized that living alone or without a partner, not being active in the labor market, less social participation, and ESR states that are typified by loneliness are associated with worse episodic memory and increased rates of episodic memory decline in older age.

Partially confirming the study’s first hypothesis, less social participation in older age was associated with worse episodic memory. This association was weak but independent of the potential negative effects of older age, having fewer years of education, economic hardship, inactivity in the labor market, worse health, and depression. In deviation to the findings of previous studies [18–20], there was no evidence that episodic memory differed significantly between older adults who lived alone and those living with at least one person in their household, nor between older adults with marital statuses that indicate either solitary states or living with a partner. This deviation from previous empirical findings could be explained by the geographical and cultural heterogeneity of the sample in the present study, as the associations of living arrangements with cognitive decline have been observed in some, but not all cultural contexts [18].

Consistent with the findings of Lara et al. [5] and McHugh et al. [15], being in ESR states in older age was associated with worse episodic memory. Confirming the study’s first hypothesis in part, older adults who were *ESR and lonely* or *ESR but not lonely* had on average worse episodic memory than those who were *neither ESR nor lonely*. Notably, the relative shortcomings in episodic memory were doubly worse among the *ESR and lonely* group than among the *ESR but not lonely* group, whereas no statistically significant differences in episodic memory emerged between the *no ESR but lonely* and the *neither ESR nor lonely* group.

These findings indicate that independent of loneliness, objective exclusionary states in older age are associated with worse episodic memory. However, when ESR and loneliness co-exist at an older age, their synergistic effect produces worse episodic memory outcomes than when ESR states are a stand-alone challenge for older adults. Hence, and consistent with the findings of previous studies examining the associations of the joint conditions of ESR and loneliness in older age [4, 23, 24], the present study provides evidence that older adults’ episodic memory suffers the most when they are challenged by a loneliness-typified ESR state.

Notwithstanding the cross-sectional associations of ESR states and episodic memory, there was no evidence that ESR states are associated with steeper episodic memory decline trajectories with aging. In contrast, there was evidence for an average and temporary increase in episodic memory among older adults who were challenged by a loneliness-typified ESR state at baseline. Although contrary to the second hypothesis of this study, this finding could be explained by the increased likelihood of transitioning out of a loneliness-typified ESR state in older age, and consequently out of an exclusionary state wherein older adults’ episodic memory suffers the most. Post-hoc cross-tabulation of the participants’ progression in and out of ESR states across the three waves confirmed that the majority (87.2%) of the older adults who were *ESR and lonely* at the first time-point of measurement transitioned out of this exclusionary state within a period of two years; whereas an additional minority (2.6%) adjusted themselves within their ESR state and did not feel lonely any longer (see Table S3 in the supplementary file). In contrast, the majority (74.2%) of older adults who were *neither ESR nor lonely* at the first time-point of measurement remained in this state after a two-year period; whereas an additional 24.4% remained socially integrated yet felt lonelier than before. Hence, the evidence of this study indicates that ESR-related shortcomings in memory may be as temporary and reversable, as loneliness-typified ESR states are in older age.

In this study, not being active in the labor market at baseline was associated with an increased rate of episodic memory decline (see Fig. 2). This finding should be interpreted through the prism of the temporal or permanent features of labor market activity in older age. Post-hoc cross-tabulation of the participants’ progression in and out of labor market activity indicates that the majority (66.9%) of those who were active in the labor market at baseline transitioned into inactivity the latest at the study’s last time-point of measurement. In contrast, the majority (91.5%) of participants who were inactive in the labor market at baseline remained so throughout the study. Given that older people who are active in the labor market tend to perform better in episodic memory tasks than those who are inactive do, the evidence of this study may indicate that transitioning out of the labor market initially has a negative effect on memory. There was no evidence, however, that labor market inactivity is associated with steeper cognitive decline trajectories in older age after older persons transition from activity to a state of non-involvement with paid labor.

The findings of this study should be considered in light of the strengths and limitations that commonly arise in longitudinal studies on cognitive aging. The attrition analysis of the study’s sample indicates that older adults who were older and worse off in terms of episodic memory, socioeconomic status, health, and social integration were underrepresented in the final sample. This may have led to an underestimation of the cognitive shortcomings of older adults who are in a disadvantaged position in these respects. The difficulties observed in the sampling capacity of large surveys to include socially excluded persons are acknowledged and may be reflected in the low representation of older person in ESR states in this study (i.e., 2.7%). Hence, the distribution of ESR states among older persons in this study may not accurately reflect the true distribution of ESR states in the older population of Europe.

A slight practice effect in the episodic memory tasks is observable in the sample, as their respective performance increased slightly from *M* = 10.51 to *M* = 10.70 correctly recalled items from wave four to wave six, decreasing thereafter to *M* = 10.19. However, the three time-points of measurement were retained in the analysis to achieve a better estimation of episodic memory slopes, although the number of these measurement points was not sufficient to estimate a potential u-shaped association between the outcome variable and the models’ predictors. Linear mixed modeling allows for the consideration of both cross-sectional and longitudinal effects on the dependent variable (here episodic memory), but the respective models do not account for the longitudinal changes in the time-variant predictors over time [44].

Future research could examine whether the transitions between ESR states in older age are associated with changes in memory at short-time intervals with a larger sample of excluded older adults. The cognitive effects stemming from transitions in and out of other exclusionary states in older age, such as living alone, living without a partner, and employment, should also be examined via similar modeling approaches. The examination of other cognitive domains in tandem, such as verbal fluency or executive function, will allow us to check for the robustness (or the specificity) of the observation made in this study in respect to the reversable nature of cognitive decline among excluded older persons.

## Conclusions

In conclusion, this study provides evidence for the benefits of an enriched social environment in supporting better cognitive outcomes in older age. Social participation and active participation in the labor market are associated with better episodic memory among older Europeans. In contrast, ESR states in older age seem to be associated with worse episodic memory, with the relevant shortcomings being worse among older adults who are challenged simultaneously by ESR and loneliness. There was no evidence for adverse longitudinal effects of ESR states on episodic memory, as any negative effects of a loneliness-typified ESR state are temporary and reversible as a function of older adults’ transition into and out of exclusionary states.

## Electronic supplementary material

Below is the link to the electronic supplementary material.

Supplementary Material 1

## Data Availability

The data that support the findings of this study are available from the Survey on Health, Aging, and Retirement in Europe SHARE (https://share-eric.eu/data/data-documentation/waves-overview/wave-8), version 8.1.0, doi: 10.6103/SHARE.w8.100. The data were used under license for the current study. The SPSS syntax file leading to the results of this study is available from the author upon reasonable request.
