# Correction: Exclusionary states in older age and their temporary effects on cognitive decline

**DOI:** 10.1186/s40359-025-02672-6

**Published:** 2025-04-08

**Authors:** Georgios Pavlidis

**Affiliations:** 1 Department of Social and Psychological Studies, Karlstad University, Universitetsgatan 2, Karlstad, 65188 Sweden; 2 Institution of Culture and Society, Linkoping University, Bredgatan 33, Norrköping, 60221 Sweden

**BMC Psychology (2025) 13:264**

10.1186/s40359-025-02574-7

Following publication of the article, it was brought to the journal’s attention that the given and family name of the author had been erroneously swapped. The order of the author’s name has since been corrected, and the corrected name may be seen in the author list of this erratum.

