# Supplementary material for: Exclusionary states in older age and their temporary effects on cognitive decline
Source: BMC Psychol. 2025 Mar 18;13:264. doi: 10.1186/s40359-025-02574-7 (PMC11917037; doi:10.1186/s40359-025-02574-7)
Supplement: Supplementary file 1 — Supplementary Material 1 [file 40359_2025_2574_MOESM1_ESM.docx]

**Table S1.** Results of the attrition analyses by conducting independent samples t-test (for continuous variables) and chi square (for categorical or ordinal variables) on demographics, EURO-D, financial distress, memory, as well as health and social network variables.

| *Descriptive statistics* | Only 4th wave | |  | All three waves | | |  |  |  | |
| --- | --- | --- | --- | --- | --- | --- | --- | --- | --- | --- |
|  | M | SD |  | M | | SD |  | df | t | |
| Age | 64.23 | 8.44 |  | 60.96 | | 5.87 |  | 33692 | 31.977* | |
| Years of education | 10.45 | 4.34 |  | 11.45 | | 4.45 |  | 33692 | -17.681* | |
| SoPa | 1.52 | 2.20 |  | 2.06 | | 2.45 |  | 33692 | -18.760* | |
| Memory | 9.26 | 3.52 |  | 10.51 | | 3.24 |  | 33692 | -28.119* | |
|  | % | |  | % | | |  |  | χ^2^ | |
| Gender (male/female) | 44.8 | 55.2 |  | 40.4 | 59.6 | |  | 1 | 47.275* | |
| Living with partner (yes/no) | 71.8 | 28.2 |  | 71.4 | 28.6 | |  | 1 | .496 | |
| Living alone (yes/no) | 20.4 | 79.6 |  | 20.1 | 79.9 | |  | 1 | .336 | |
| Depression (yes/no) | 26.9 | 73.1 |  | 21.6 | | 78.1 |  | 1 | 86.228* | |
| Gali (yes/no) | 45.4 | 54.6 |  | 37.3 | | 62.7 |  | 1 | 161.051* | |
| Active in labour (yes/no) | 26.6 | 73.4 |  | 39.2 | | 60.8 |  | 1 | 455.336* | |
| Perceived health |  |  |  |  | |  |  |  |  | |
| Excellent | 7.7 | |  | 10.4 | | |  | 4 | 604.183* | |
| Very good | 17.6 | |  | 25.6 | | |  |  |  | |
| Good | 37.3 | |  | 39.8 | | |  |  |  | |
| Fair | 26.7 | |  | 19.3 | | |  |  |  | |
| Poor | 10.6 | |  | 4.9 | | |  |  |  | |
| Financial distress |  |  |  |  | |  |  | 3 | 274.603* | |
| Easily | 27.2 | |  | 35.4 | | |  |  |  | |
| Fairly easily | 32.0 | |  | 32.6 | | |  |  |  | |
| With some difficulty | 28.9 | |  | 24.3 | | |  |  |  | |
| With great difficulty | 11.8 | |  | 7.7 | | |  |  |  | |
| ESR states |  | |  |  | | |  | 3 | 16.005* | |
| ESR not lonely | 1.8 | |  | 1.7 | | |  |  |  | |
| ESR and lonely | 1.5 | |  | 1.0 | | |  |  |  | |
| Neither ESR nor lonely | 63.4 | |  | 65.1 | | |  |  |  | |
| Not ESR but lonely | 33.4 | |  | 32.2 | | |  |  |  | |
| *Note.* M = mean, SD = standard deviation, df = degrees of freedom, χ^2^ = chi-square, R-UCLA = loneliness scale, SoPA = social participation, Gali = limitations due to health, ESR = states of exclusion from social relations, namely coring zero in network size; lonely are those with R-UCLA scores >3, not lonely those with R-UCLA scores = 3.  * *p* < .001 | | | | | | | | | |  |

**Table S2**. Linear mixed effects model (Model 3) predicting cross-sectionally the aggregated episodic memory scores (immediate and delayed recall) across three waves, with demographics, health, social participation, and states of exclusion from social relations as predictors.

| *Parameter* | *Estimate* | *S.E* | *df* | *t* | *Sig.* | *95% CI* | |
| --- | --- | --- | --- | --- | --- | --- | --- |
|  |  |  |  |  |  | *Lower* | *Upper* |
| Time | -.167 | .020 | 16350.468 | -8.500 | <.001 | -.205 | -.128 |
| Age ^(fourth wave)^ | -.107 | .005 | 9937.560 | -20.871 | <.001 | -.117 | -.097 |
| Gender _(male/female)_ | .957 | .057 | 7795.524 | 16.928 | <.001 | .847 | 1.068 |
| Education | .120 | .006 | 7909.414 | 19.039 | <.001 | .107 | .132 |
| Living alone _(yes/no)_ | -.123 | .078 | 21008.048 | -1.577 | .115 | -.276 | .030 |
| Financial distress | -.269 | .024 | 23106.881 | -11.305 | <.001 | -.316 | -.223 |
| Living with partner _(yes/no)_ | .009 | .075 | 16697.501 | .123 | .902 | -.138 | .157 |
| Active in labour _(yes/no)_ | -.011 | .051 | 22107.297 | -.215 | .829 | -.111 | .089 |
| Gali _(yes/no)_ | .057 | .043 | 21931.484 | 1.317 | .188 | -.028 | .142 |
| Perceived health | -.318 | .024 | 23059.587 | -13.234 | <.001 | -.365 | -.271 |
| Depression _(yes/no)_ | .399 | .049 | 21928.478 | 8.094 | <.001 | .303 | .496 |
| Social participation | .126 | .009 | 22718.932 | 14.363 | <.001 | .109 | .144 |
| ESR^1^ |  |  |  |  |  |  |  |
| ESR not lonely | -.560 | .171 | 20033.404 | -3.280 | .001 | -.894 | -.225 |
| ESR and lonely | -.536 | .183 | 20526.992 | -2.937 | .003 | -.894 | -.178 |
| Not ESR but lonely | -.178 | .042 | 22265.394 | -4.231 | <.001 | -.261 | -.096 |
| *Note.* SE = Standard error of means, df = degrees of freedom, CI = confidence intervals, Gali = limitations due to health, ESR = states of exclusion from social relations, namely coring zero in network size; lonely are those with R-UCLA scores >3, not lonely those with R-UCLA scores = 3  ^1^ Reference group = neither ESR nor lonely | | | | | | | |

**Table S3.** Cross-tabulation on the progression of states of exclusion from social relations in waves four and six, and in waves six and eight

|  | | | ESR _Wave 6_ | | | | Total |
| --- | --- | --- | --- | --- | --- | --- | --- |
|  |  |  | ESR not lonely | ESR and lonely | no ESR nor lonely | no ESR but lonely |  |
| ESR _Wave 4_ | ESR not lonely | Count | 5 | 7 | 81 | 40 | 133 |
|  |  | % _within Wave 4_ | 3.8% | 5.3% | 60.9% | 30.1% | 100.0% |
|  | ESR and lonely | Count | 2 | 8 | 27 | 41 | 78 |
|  |  | % _within Wave 4_ | 2.6% | 10.3% | 34.6% | 52.6% | 100.0% |
|  | no ESR nor lonely | Count | 42 | 33 | 3782 | 1243 | 5100 |
|  |  | % _within Wave 4_ | 0.8% | 0.6% | 74.2% | 24.4% | 100.0% |
|  | no ESR but lonely | Count | 18 | 47 | 1013 | 1441 | 2519 |
|  |  | % _within Wave 4_ | 0.7% | 1.9% | 40.2% | 57.2% | 100.0% |
| Total | | Count | 67 | 95 | 4903 | 2765 | 7830 |
|  |  | % _within Wave 4_ | 0.9% | 1.2% | 62.6% | 35.3% | 100.0% |

|  | | | ESR _Wave 8_ | | | | Total |
| --- | --- | --- | --- | --- | --- | --- | --- |
|  |  |  | ESR not lonely | ESR and lonely | no ESR nor lonely | no ESR but lonely |  |
| ESR _Wave 6_ | ESR not lonely | Count | 7 | 1 | 42 | 17 | 67 |
|  |  | % _within Wave 6_ | 10.4% | 1.5% | 62.7% | 25.4% | 100.0% |
|  | ESR and lonely | Count | 2 | 8 | 28 | 57 | 95 |
|  |  | % _within Wave 6_ | 2.1% | 8.4% | 29.5% | 60.0% | 100.0% |
|  | no ESR nor lonely | Count | 28 | 12 | 3807 | 1056 | 4903 |
|  |  | % _within Wave 6_ | 0.6% | 0.2% | 77.6% | 21.5% | 100.0% |
|  | no ESR but lonely | Count | 13 | 32 | 1017 | 1703 | 2765 |
|  |  | % _within Wave 6_ | 0.5% | 1.2% | 36.8% | 61.6% | 100.0% |
| Total | | Count | 50 | 53 | 4894 | 2833 | 7830 |
|  |  | % _within Wave 6_ | 0.6% | 0.7% | 62.5% | 36.2% | 100.0% |

*Note.* ESR = states of exclusion from social relations, namely network size = 0; lonely are those with R-UCLA scores >3, not lonely those with R-UCLA scores = 3, R-UCLA = Revised UCLA loneliness scale
